# Urate inhibits microglia activation to protect neurons in an LPS-induced model of Parkinson’s disease

**DOI:** 10.1186/s12974-018-1175-8

**Published:** 2018-05-02

**Authors:** Li-Hui Bao, Ya-Nan Zhang, Jian-Nan Zhang, Li Gu, Hui-Min Yang, Yi-Ying Huang, Ning Xia, Hong Zhang

**Affiliations:** Department of Neurobiology, School of Basic Medical Sciences, Beijing Institute for Brain Disorders and Key Laboratory for Neurodegenerative Disorders of the Ministry of Education, Capital Medical University, Beijing, 100069 China

**Keywords:** Urate, Microglia, Inflammation, Urate transporter, Parkinson’s disease

## Abstract

**Background:**

Multiple risk factors contribute to the progression of Parkinson’s disease, including oxidative stress and neuroinflammation. Epidemiological studies have revealed a link between higher urate level and a lower risk of developing PD. However, the mechanistic basis for this association remains unclear. Urate protects dopaminergic neurons from cell death induced by oxidative stress. Here, we investigated a novel role of urate in microglia activation in a lipopolysaccharide (LPS)-induced PD model.

**Methods:**

We utilized Griess, ELISA, real-time PCR, Western blot, immunohistochemistry, and immunofluorescence to detect the neuroinflammation. For Griess, ELISA, Western blot, and immunofluorescence assay, cells were seeded in 6-well plates pre-coated with poly-l-lysine (PLL) and incubated for 24 h with the indicated drugs. For real-time PCR assay, cells were seeded in 6-well plates pre-coated with PLL and incubated for 6 h with the indicated drugs. For animal experiments, rats were injected with urate or its vehicle twice daily for five consecutive days before and after stereotaxic surgery. Rats were killed and brain tissues were harvested after 4 weeks of LPS injection.

**Results:**

In cultured BV2 cells and rat primary microglia, urate suppressed proinflammatory cytokine production and inducible cyclooxygenase 2 and nitric oxide synthase expression to protect dopaminergic neurons from the toxic effects of activated microglia. The neuroprotective effects of urate may also be associated with the stimulation of anti-inflammatory factors interleukin 10 and transforming growth factor β1. Intracellular urate level was increased in a dose-dependent manner upon co-treatment with urate and LPS as compared with LPS alone, an effect that was abrogated by pretreatment with probenecid (PBN), an inhibitor of both glucose transporter 9 and urate transporter 1 (URAT1). PBN also abolished the anti-inflammatory effect of urate. Consistent with these in vitro observations, the number of tyrosine hydroxylase-positive neurons was decreased and the loss of motor coordination was reversed by urate administration in an LPS-induced rat model of PD. Additionally, increased plasma urate level abolished the reduction of URAT1 expression, the increase in the expression of interleukin-1β, and the number of ionized calcium-binding adaptor molecule 1-positive microglia along with changes in their morphology.

**Conclusions:**

Urate protects neurons against cytotoxicity induced by microglia activation via modulating urate transporter-mediated intracellular urate level.

**Electronic supplementary material:**

The online version of this article (10.1186/s12974-018-1175-8) contains supplementary material, which is available to authorized users.

## Background

Parkinson’s disease (PD) is a progressive neurodegenerative disorder characterized by bradykinesia, resting tremor, cogwheel rigidity, and postural instability due to the loss of dopaminergic (DA) neurons in the substantia nigra (SN) pars compacta and the formation of Lewy bodies and Lewy neurites in surviving neurons. Microglia activation and the consequent neuroinflammation play important roles in PD progression. Autopsies of PD patients have revealed DA neuron loss accompanied by microglia activation and the release of large amounts of nitric oxide (NO), tumor necrosis factor (TNF)-α, interleukin (IL)-1β, and other proinflammatory cytokines [1–3]. Experimental studies have confirmed that inhibiting microglia activation blocks DA neuron degeneration in lipopolysaccharide (LPS)-induced cells [4] and animal models of PD [5]. It is thought that endotoxins (e.g., LPS) [6] induce microglia activation and production of pro-inflammatory factors and chemokines, leading to neurodegeneration and further activation of microglia, which is known as a perpetuating cycle of neurotoxicity and microglia activation to aggravate neurodegenerative diseases [7]. Controlling microglia activation and the resultant release of neurotoxins can thus have neuroprotective effects.

Urate (2, 6, 8-trioxy-purine) is an end product of purine metabolism in humans that is known to have protective effects in various nervous system diseases [8–10]. Urate level is considered as a biomarker for PD risk that has therapeutic potential [9]. Epidemiological studies have reported a correlation between urate level and a lower risk of developing PD as well as slower disease progression [11–13]. Accordingly, lower urate concentration has been detected in the serum and SN of PD patients [14]. Urate has been shown to confer neuroprotection in various cells and animal models of PD by abrogating the neurotoxic effects of 6-hydroxydopamine (6-OHDA) on DA neurons in the rat nigrostriatal pathway [15]. It has also been shown to protect cultured spinal cord and hippocampal neurons from glutamate-induced excitotoxicity [16] and peroxynitrite-induced cell death [17]. Mice lacking the urate oxidase gene have elevated the level of urate in the brain and are resistant to the negative effects of 6-OHDA on SN DA neuron number, striatal DA content, and motor behavior [18]. It was also reported that urate acts as a natural antioxidant to eliminate superoxide anion and hydroxyl radical in the blood and brain [19, 20] and inhibits lipid peroxidation and DNA damage induced by free radicals [21]. Also, urate enhances astrocytic glutathione synthesis and release [22]. Interestingly, it has been shown that application of urate prevents liver neutrophil infiltration and injury during hemorrhagic shock [23] and inhibited tyrosine nitration to preserve the integrity of the blood–brain barrier by blocking the entry of inflammatory cells into the central nervous system (CNS) [24], suggesting that urate might play a role in modulating neuroinflammation to protect from neuron damage.

In this study, we investigated the role of urate in LPS-induced microglia activation in vitro and in vivo. We found that urate suppressed LPS-induced activation of microglia and thereby prevented neuronal death. These findings indicate that suppressing neuroinflammation with urate can be an effective treatment for PD.

## Methods

### Cell culture and drug treatments

BV2 murine microglia cells were kindly provided by Professor Xiao-Min Wang (Capital Medical University, China) and MN9D dopaminergic neuronal cells were a generous gift from Professor Hui Yang (Capital Medical University, Beijing, China). BV2 cells and MN9D cells were cultured in Dulbecco’s Modified Eagle’s Medium (DMEM/F-12) with 10% fetal bovine serum and 1% penicillin/streptomycin (Life Technologies, Carlsbad, CA, USA). The cells were maintained in a humidified incubator with 95% air in 5% CO_2_ at 37 °C. Cells were seeded in 6-well plates for experiments at 80% confluency.

Rat primary microglia were obtained from 0- to 24-h-old Sprague-Dawley rats (Beijing Weitong Lihua Laboratory Animal Center, Beijing, China; SCXK 2012-0001) according to a previously published protocol [25], with minor modifications. Briefly, animals were deeply anesthetized and their brains were dissected. The meninges, choroid plexus, brainstem, and cerebellum were removed carefully. Brains were transferred to a 50-ml centrifuge tube containing 5 ml Hank’s Balanced Salt Solution. The tissue was dissociated by trituration with a pipette and the cell suspension was filtered through a 40-μm pore nylon strainer. Samples were centrifuged for 10 min at 1000 rpm. The supernatant was discarded, and the pellet was resuspended in warm DMEM/F12. The cells were then transferred to a 75-cm^2^ flask coated with poly-l-lysine (PLL; Sigma-Aldrich, St. Louis, MO, USA) and incubated at 37 °C and 5% CO_2_. Half of the culture medium was changed every 3 days. After 14 days of culture, primary microglial cells were harvested by shaking the flask for 2 h at 180 rpm and then seeding the cells on new PLL-coated plates. To assess the purity of the culture, cells were immunolabeled with an antibody against ionized calcium-binding adapter molecule (Iba)-1 (1:500; Wako Pure Chemical Industries, Osaka, Japan); over 96% of the cells were immunopositive. Urate (Sigma-Aldrich; prepared as a 1000× stock solution by dissolving in 1 M NaOH) was applied to microglia for 30 min prior to treatment with LPS (Sigma-Aldrich; dissolved in double-distilled water). Probenecid (PBN) (Sigma-Aldrich; prepared as a 1000× stock solution containing 1 M NaOH) was administered 30 min prior to urate treatment. NaOH had no effect on the function of urate (Additional file 1: Figure S1). BV2 cells and primary microglia were treated under the indicated conditions to measure the inflammatory factors. Then supernatants were collected as conditioned medium continued to co-culture with MN9D cells to examine neuroprotection by measuring viability of MN9D cells.

### Measurement of nitrite level

Nitrite release was measured as an indicator of NO production. BV2 cells (4.0 × 10^5^/well) or primary rat microglia (1.0 × 10^6^/well) were seeded in 6-well plates pre-coated with PLL and incubated for 24 h with the indicated drugs. The nitrite concentration in the culture supernatant was evaluated with a Griess kit (Promega, Madison, WI, USA) according to the manufacturer’s instructions. The absorbance at 540 nm was measured on a microplate reader (Elx800; Bio-Tek Instruments, Winooski, VT, USA).

### Enzyme-linked immunosorbent assay (ELISA) measurement

BV2 cells (4.0 × 10^5^/well) or primary rat microglia (1.0 × 10^6^/well) were seeded in 6-well plates pre-coated with PLL and incubated for 24 h with indicated drugs. IL-1β and IL-10, TNF-α, prostaglandin (PG)E2, and transforming growth factor (TGF)-β1 levels in the medium were measured with ELISA kits (ExCell Bio, Shanghai, China) according to the manufacturer’s protocol. The absorbance at 450 nm was measured on a microplate reader.

Tissues were homogenized in ice-cold tissue lysis buffer containing 137 mM NaCl, 20 mM Tris (pH 8.0), 1% (*v*/*v*) glycerol, 1% (*v*/*v*) Nonidet P-40 (NP40), 1 mM phenylmethylsulfonyl fluoride, and 0.5 mM sodium vanadate. The homogenate was centrifuged at 1500×*g* for 15 min at 4 °C. The levels of TNF-α were detected using rat TNF-α enzyme-linked immunosorbent assay (ELISA) kits (Shanghai ExCell Biology Inc., Shanghai, China), according to the manufacturer’s instructions. The absorbance at 450 nm was measured on a microplate reader.

### Protein extraction and Western blotting

Cells were washed three times with cold phosphate-buffered saline (PBS) and lysed in lysis buffer. Tissues were lysed with RIPA lysis buffer (Solarbio, Beijing, China). The protein concentration was determined with a bicinchoninic acid assay kit (Thermo Fisher Scientific, Rockford, IL, USA) according to the manufacturer’s instructions. Proteins were separated by sodium dodecyl sulfate–polyacrylamide gel electrophoresis and then transferred to a polyvinylidene difluoride membrane that was blocked with 10% skim milk at room temperature for 1 h and then probed overnight at 4 °C with primary antibodies against cyclooxygenase (COX)-2 (1:1000, Cell Signaling Technology, Danvers, MA, USA), inducible nitric oxide synthase (iNOS) (1:250), and glucose transporter member (Glut) 9 (1:800) (both from Abcam, Cambridge, MA, UK) and urate transporter (URAT) 1 (1:500, Proteintech, Rosemont, IL, USA), IL-1β (1:400, R&D Systems, Minneapolis, MN, USA), and GAPDH (1:1000, Cell Signaling Technology, Danvers, MA, USA). The following day, the membrane was washed three times with Tris-buffered saline with Tween-20 and then incubated with the appropriate horseradish peroxidase-conjugated secondary antibody (Cell Signaling Technology) for 1 h at room temperature. Immunoreactivity was visualized by enhanced chemiluminescence (Millipore Corporation, Billerica, MA, USA), and the signal intensity was quantified using ImageJ software (National Institutes of Health, Bethesda, MD, USA).

### Real-time PCR analysis

BV2 cells (4.0 × 10^5^/well) or primary rat microglia (1.0 × 10^6^/well) were seeded in 6-well plates and treated with the indicated drugs for 6 h. Total RNA was extracted using an RNeasy kit (Qiagen, Duesseldorf, Germany), and 1 μg was reverse transcribed into cDNA using the ImProm-II Reverse Transcription System (Promega, Madison, WI, USA) in a total volume of 20 μl. TNF-α, IL-1β, β-actin and glyceraldehyde 3-phosphate dehydrogenase (GAPDH) genes were amplified using the forward and reverse primers (200 nM each) listed in Table 1 along with 20 ng cDNA template and 10 μl SYBR FAST qPCR Kit Master Mix (2×) (Kapa Biosystems, Wilmington, MA, USA) in a total reaction volume of 20 μl. Amplification was performed over 40 cycles of 95 °C for 3 s and 60 °C for 30 s on a CFX96 real-time PCR detection system (Bio-Rad, Hercules, CA, USA). Target gene expression levels were normalized to that of GAPDH using the data analysis software provided with the system.

**Table 1:** Real-time PCR primers (F, forward primer; R, reverse primer) and size of amplicon

Primer	Forward (5′-3′)	Reverse (5′-3′)
Mouse IL-1β	CTgTgTCTTTCCCgTggACC	CAgCTCATATgggTCCgACA
Mouse TNF-α	CAGCCGATGGGTTGTACCTT	TGTGGGTGAGGAGCACGTAGT
Mouse β-actin	TgCTgTCCCTgTATgCCTCT	TTgATgTCACgCACgATTTC
Rat IL-1β	TCTGTGACTCGTGGGATGAT	GGAGAATACCACTTGTTGGC
Rat TNF-α	ACTCCCAGGTTCTCTTCAAG	CAGAGAGGAGGCTGACTTTC
Rat GAPDH	TGACATCAAGAAGGTGGTGAAGC	GGAAGAATGGGAGTTGCTGTTG

TNF-α, IL-1β, β-actin, and GAPDH genes were amplified using the forward and reverse primers listed in the above

### Measurement of urate level

For in vitro experiments, BV2 cells (1.0 × 10^6^/well) were seeded in 100-mm culture dishes pre-coated with PLL. Once they reached 70–80% confluence, the cells were treated with the indicated drugs for 24 h, washed three times with PBS, and lysed in urate assay buffer. For in vivo experiments, 10 days after urate injection and 4 weeks after LPS injection, blood was collected via the caudal vein into an anticoagulant tube within 1 h of the final injection. The blood was centrifuged for 10 min at 800×*g*, and the plasma was transferred to tubes and stored at − 80 °C until use. Intracellular and plasma urate concentration was measured with a urate colorimetric/fluorometric assay kit (BioVision, Milpitas, CA, USA) according to the manufacturer’s protocol. The absorbance at 570 nm was determined on a microplate reader.

### MTS assay

Cell viability was evaluated with the 3-(4, 5-dimethylthiazol-2-yl)-5-(3-carboxymethoxyphenyl)-2-(4-sulfophenyl)-2H-tetrazolium (MTS) assay (Cell Tilter 96 Aqueous Assay; Promega). Cells were seeded on PLL-coated 96-well plates (8.0 × 10^3^ cells /well). Once they reached 70–80% confluence, the cells were treated with conditioned medium or the indicated drugs. After 24 h, MTS solution was added followed by incubation for 1 h at 37 °C, and the absorbance at 490 nm was measured on a microplate reader.

### Animals and treatment

Male Sprague-Dawley rats (5-week-old, weighing 180–220 g) were housed in cages under standard laboratory conditions at 20–22 °C on a 12:12-h light/dark cycle with free access to food and tap water. Animals were maintained in the housing facilities for at least 1 week prior to experiments. All procedures were performed in accordance with the guidelines of the Animal Care and Use Committee of Capital Medical University, Beijing, China (2006–0009), and conformed to the Guide for the Care and Use of Laboratory Animals published by the National Institutes of Health (NIH Publications No. 8023, revised 1978).

The sample sizes used in this study were based on estimations by a power analysis. Rats (*n* = 45) were randomly divided into four groups: sham (PBS + intraperitoneal [i.p.] injection of vehicle; *n* = 12), LPS (LPS + i.p. injection of vehicle; *n* = 14), LPS + urate (LPS + i.p. injection of urate; *n* = 15), and urate (i.p. injection of urate; *n* = 4). Rats were injected with 200 mg/kg urate (40 mg/ml, dissolved in 0.9% NaCl solution) or vehicle twice daily, with 1 h between two injections. Rats were anesthetized by intraperitoneal injection of chloral hydrate (300 mg/kg; Tianjin Guang Fu Fine Chemical Research Institute, Tianjin, China) and placed in a stereotaxic apparatus. LPS (5 μg/μl in PBS, for a total dose of 10 μg/300 g) was injected into the right SN (5.5 mm posterior and 1.5 mm lateral to bregma, and 8.3 mm down from the dural surface) at a flow rate of 0.5 μl/min. The needle was kept in place for over 5 min before slow retraction to prevent reflux along the injection tract. The mortality rate of rats in the LPS injection group was < 10%. Rats were sacrificed by anesthetization with chloral hydrate followed by decapitation 4 weeks after LPS administration.

### Behavioral tests

The rotarod test was carried out at three time points: before drug treatment and at 3 and 4 weeks after the surgery. Rats were tested under accelerating rotor mode (constant acceleration from 5 to 40 rpm for 2 min). The length of time that the animal remained on the rod was noted.

### Immunohistochemical and immunofluorescence analyses

Animals were anesthetized with chloral hydrate and then transcardially perfused with 4% paraformaldehyde (pH 7.4). The brains were removed and post-fixed overnight in the same solution then sequentially placed in 15 and 30% sucrose at 4 °C. Serial coronal sections were cut at a thickness of 40 μm on a freezing microtome, and a 1:6 series of sections was used for quantitative analysis as previously described (Xia et al., 2015). Sections were permeabilized for 20 min with PBS containing 0.3% Triton X-100 and incubated overnight at 4 °C with mouse anti-tyrosine hydroxylase (TH, 1:5000; Sigma-Aldrich) and rabbit anti-Iba-1 (1:500; Wako Pure Chemical Industries) antibodies. An ABC kit (Vector Laboratories, Burlingame, CA, USA) was used for immunohistochemical detection. For immunofluorescence labeling, sections were incubated with tetramethylrhodamine- or fluorescein isothiocyanate-conjugated goat anti-mouse or anti-rabbit antibodies (ZSGB-BIO, Beijing, China; both 1:100) for 1 h. Quantification of TH-positive cells in SN was counted by sterology using Stereo Investigator software (MBF Bioscience, USA). TH-positive fibers in STR and Iba-1 positive cells in the striatum and SN were performed using Image Pro Plus v5.0 image analysis software (Datacell, UK).

### Statistical analysis

Data are expressed as the mean ± SD and were analyzed using Prism 5.0 software (GraphPad Inc., La Jolla, CA, USA). Where parametric tests were used, we checked normal distribution and difference in variance by the Shapiro–Wilk test and an *F* test, respectively. One-way ANOVA followed by Dunnett’s test was used for multiple-group comparisons. At least three independent experiments were performed for each assay. *p* < 0.05 was considered significant throughout the study.

## Results

### Urate suppressed LPS-induced microglia activation in BV2 cells

**Fig. 1:**
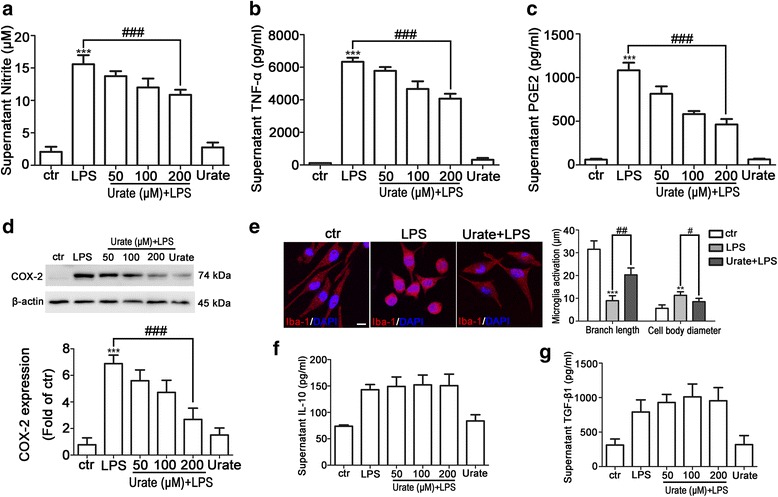
Urate inhibits LPS-induced microglia activation in BV2 cells. **a**–**c** BV2 cells were pretreated for 30 min with 50, 100, or 200 μM urate followed by 100 ng/ml LPS for 24 h; NO (**a**, *n* = 4), TNF-α (**b**, *n* = 5), and PGE2 (**c**, *n* = 3) levels in the culture supernatant were measured by ELISA. **d** BV2 cells were pretreated with indicated concentrations of urate and LPS, and COX-2 level was detected by Western blotting (up, *n* = 4). Protein band intensity was normalized to β-actin and is expressed as fold difference relative to the control group (down). **e** BV2 cells were pretreated with 200 μM urate followed by LPS, and microglia activation was evaluated by immunofluorescence detection of Iba-1 (red) and cell nuclei were stained with DAPI (blue) (left, *n* = 3). Branch length and cell body diameter were quantified with ImageJ software (right). Scale bar = 10 μm. **f**, **g** BV2 cells were pretreated for 30 min with indicated concentrations of urate followed by LPS for 24 h; IL-10 (**f**, *n* = 4) and TGF-β1 (**g**, *n* = 3) levels in the culture supernatant were measured by ELISA. Untreated cells served as the control (ctr). Data represent the mean ± SD. ^**^p < 0.01, ^***^p < 0.001 vs. control group; ^#^*p* < 0.05, ^##^*p* < 0.01, ^###^*p* < 0.001 vs. LPS group (one-way analysis of variance)

Microglia release proinflammatory cytokines and also produce anti-inflammatory factors in response to LPS stimulation [26, 27]. To investigate the effect of urate on LPS-induced toxicity in microglia, BV2 cells were pretreated with urate prior to exposure to LPS for 24 h. NO, TNF-α, and PGE2 levels were markedly upregulated by LPS treatment, whereas urate dose-dependently inhibited the release of proinflammatory factors, NO from 11.7% (*p* = 0.0057) to 30.0% (*p* < 0.0001), TNF-α from 8.8% (*p* = 0.0028) to 35.8% (*p* < 0.0001), and PGE2 from 24.8% (*p* < 0.0001) to 57.3% (*p* < 0.0001) (Fig. 1a–c). Inducible COX-2 is an important contributor to neuroinflammatory disease, while PGE2 is synthesized through the activity of COX-2 [28]. COX-2 expression was upregulated after LPS treatment whereas urate reversed this effect in a dose-dependent manner (Fig. 1d). Activated microglia undergo significant changes in morphology, from a small cell body with long branches to a round shape with short branches [29]. Immunofluorescence detection of Iba-1 revealed that LPS stimulation decreased the length of cell branches (from 30.00 ± 3.135 μm to 8.942 ± 0.8293 μm, *p* < 0.0001) and increased cell body diameter (from 5.600 ± 0.7627 μm to 11.32 ± 0.5922 μm, *p* = 0.0002) as compared to the control group; these changes were reversed (length of cell branches from 8.942 ± 0.8293 μm to 20.30 ± 1.518 μm, *p* < 0.0001; cell body diameter from 11.32 ± 0.5922 μm to 8.531 ± 0.7181, *p* = 0.0171) by urate treatment (Fig. 1e). To determine whether urate affected the production of anti-inflammatory factors, we measured the levels of IL-10 and TGF-β1 by ELISA. Urate slightly increased the levels of IL-10 and TGF-β1 induced by LPS in BV2 cell cultures, although the difference relative to the LPS treatment was non-significant (*p* = 0.4158, *p* = 0.2282) (Fig. 1f, g). The proliferation of BV2 cells was not altered significantly by treatment under the indicated conditions (Additional file 1: Figure S1). These data indicated that urate played a protective role against inflammation induced by microglia activation in BV2 cells.

### Urate suppressed LPS-induced microglia activation in rat primary microglia

**Fig. 2:**
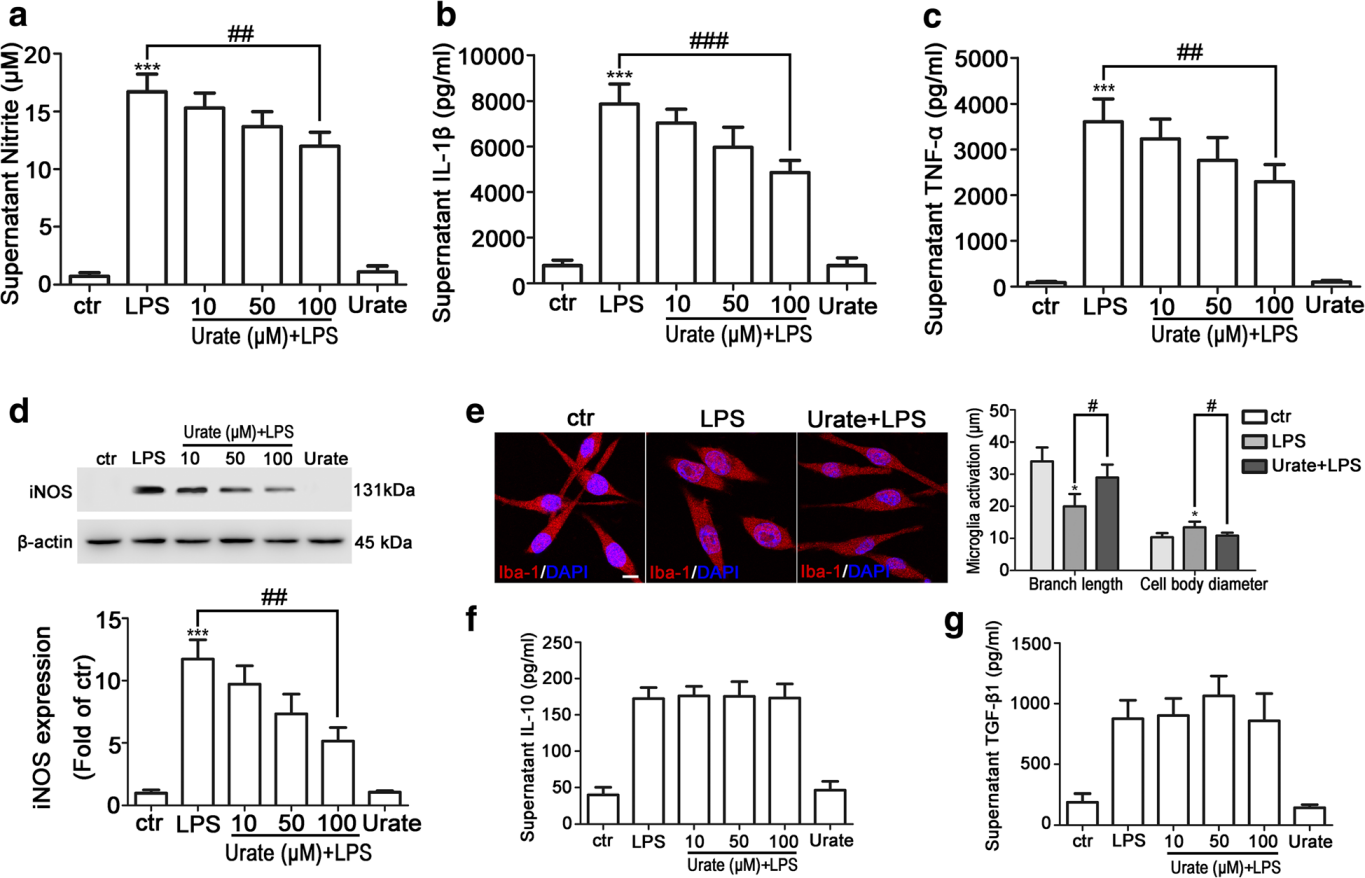
Urate inhibits LPS-induced activation of rat primary microglia. **a**–**c** Primary microglia were pretreated for 30 min with 10, 50, or 100 μM urate followed by 10 ng/ml LPS for 24 h; NO (**a**, *n* = 4), IL-1β (**b**, *n* = 3), and TNF-α (**c**, *n* = 3) levels in the culture supernatant were measured by ELISA. **d** Primary microglia were pretreated with indicated concentrations of urate followed by LPS, and iNOS level was detected by Western blotting (up, *n* = 4). Protein band intensity was normalized to β-actin and is expressed as fold difference relative to the control group (down). **e** Primary microglia were pretreated with 100 μM urate followed by LPS, and microglia activation was evaluated by immunofluorescence detection of Iba-1 (red) and cell nuclei were stained with DAPI (blue) (left, *n* = 3). Branch length and cell body diameter were quantified with ImageJ software (right). Scale bar = 10 μm. **f**, **g** Primary microglia cells were pretreated for 30 min with indicated concentrations of urate followed by LPS for 24 h; IL-10 (**f**, *n* = 4) and TGF-β1 (**g**, *n* = 4) levels in the culture supernatant were measured by ELISA. Untreated cells served as a control (ctr). Data represent the mean ± SD. ^*^p < 0.05  ^***^p < 0.001 vs. control group; ^#^*p* < 0.05, ^##^*p* < 0.01, ^###^*p* < 0.001 vs. LPS group (one-way analysis of variance)

The anti-inflammatory effect of urate on LPS-induced microglia activation was also examined in rat primary microglia. Treatment with LPS for 24 h stimulated the release of NO, IL-1β, and TNF-α, whereas urate dose-dependently suppressed the production of proinflammatory cytokines induced by LPS, NO from 8.3% (*p* = 0.0516) to 28.2% (*p* < 0.0001), TNF-α from 10.4% (*p* = 0.1961) to 36.4% (*p* = 0.0004), and IL-1β from 10.6% (*p* = 0.0835) to 38.2% (*p* < 0.0001) (Fig. 2a–c). NO is produced by iNOS and contributes to microglia activation [30, 31]. We therefore examined the effect of urate on iNOS expression in LPS-stimulated rat primary microglia. After 24 h of LPS treatment, iNOS expression was increased; however, this was abrogated in a dose-dependent manner by urate treatment (Fig. 2d). Primary microglia exhibited morphological changes that were comparable to those observed in BV2 cells: the length of branches was decreased (from 34 ± 2.717 μm to 19.97 ± 1.950 μm, *p* < 0.0053) and cell body diameter was increased (from 10.36 ± 0.5732 μm to 13.45 ± 0.8809 μm, *p* = 0.0184) in the LPS-treated as compared to the control group, which was reversed (branches length from 19.97 ± 1.950 μm to 28.97 ± 2.024 μm, *p* = 0.0186; cell body diameter from 13.45 ± 0.8809 μm to 10.86 ± 0.4175 μm, *p* = 0.0243) in the presence of urate (Fig. 2e). In addition, similar effect of urate on the levels of IL-10 and TGF-β1 induced by LPS was observed in rat primary microglia (Fig. 2f, g). The proliferation of primary microglia was not altered significantly by treatment under the indicated conditions (Additional file 2: Figure S2). Taken together, the data indicated that urate played a consistent anti-inflammatory role in primary microglia as in BV2 cells.

### Urate suppressed proinflammatory cytokine gene expression in microglia

**Fig. 3:**
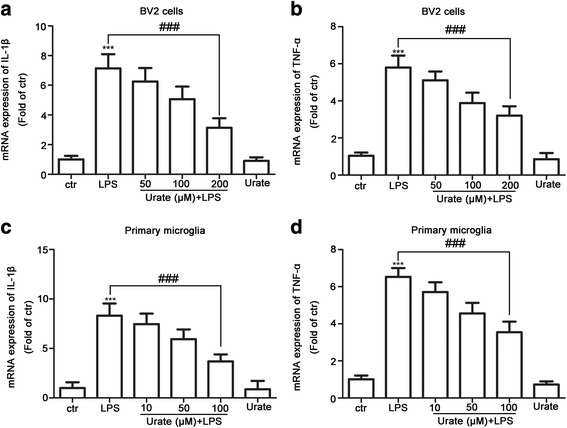
Urate suppresses proinflammatory cytokine expression in BV2 cells and rat primary microglia. **a**–**d** BV2 cells (**a**, **b**; *n* = 4) and primary microglia (**c**, **d**; *n* = 4) were pretreated for 30 min with indicated concentrations of urate followed by LPS for 6 h. IL-1β and TNF-α transcription levels were analyzed by quantitative real-time PCR and normalized to that of GAPDH. Untreated cells served as a control (ctr). Data represent the mean ± SD. ^***^p < 0.001 vs. control group; ^###^*p* < 0.001 vs. LPS group (one-way analysis of variance)

LPS induces the expression of IL-1β and TNF-α in microglia [32]. To further determine whether urate modulated the levels of proinflammatory cytokines, we examined IL-1β and TNF-α expression by quantitative real-time PCR analysis. IL-1β and TNF-α levels were elevated in BV2 cells by treatment with LPS relative to the control group, an effect that was abrogated by pretreatment with urate (Fig. 3a, b). A similar anti-inflammatory effect was observed in primary microglia treated with LPS and urate (Fig. 3c, d). These results indicated that urate suppressed proinflammatory cytokine gene expression in both BV2 cells and rat primary microglia.

### Intracellular accumulation of urate was required for its anti-inflammatory effect in microglia

**Fig. 4:**
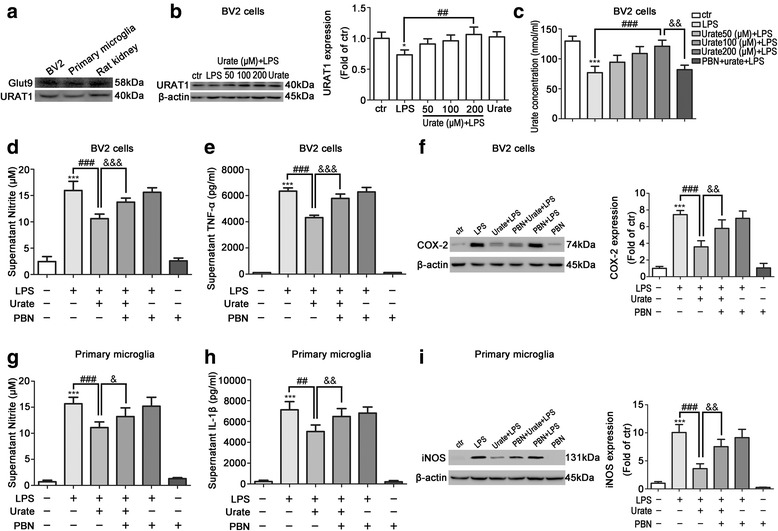
Intracellular urate accumulation is required for its anti-inflammatory effect in microglia. **a** BV2 cells and rat primary microglia seeded in a 6-well plate were cultured for 24 h; Glut9 and URAT1 levels were evaluated by Western blotting (*n* = 3). Protein extract from rat kidney tissue was probed as positive control. **b** BV2 cells were pretreated for 30 min with indicated concentrations of urate followed by LPS treatment for 24 h. Cell lysates were probed for URAT1 using indicated specific antibodies (left, *n* = 3). Protein band intensity was normalized to β-actin and is expressed as fold difference relative to the control group (right). **c** BV2 cells were pretreated with 1000 μM PBN and urate followed by LPS. Urate concentration in cell lysates was measured with a fluorometric assay kit (*n* = 6). **d**–**f** BV2 cells were pretreated with PBN and urate followed by LPS; NO (**d**, *n* = 4) and TNF-α (**e**, *n* = 4) levels in the culture supernatant were detected by ELISA. COX-2 expression was evaluated by Western blotting relative to the level of β-actin (**f**, left, *n* = 4). Protein band intensity was normalized to β-actin and is expressed as fold difference relative to the control group (**f**, right). **g**–**i** Rat primary microglia were pretreated with PBN and urate followed by LPS; NO (**g**, *n* = 4) and IL-1β (**h**, *n* = 3) levels in the culture supernatant were detected by ELISA. iNOS level was evaluated by Western blotting (**i**, left, *n* = 4) relative to that of β-actin and is expressed as fold difference relative to control group (**i**, right). Untreated cells served as the control (ctr). Data represent the mean ± SD. ^*^p < 0.05, ^***^p < 0.001 vs. control group; ^##^*p* < 0.01, ^###^*p* < 0.001 vs. LPS group; ^&^*p* < 0.05, ^&&^*p* < 0.01, ^&&&^*p* < 0.001 vs. urate+LPS group (one-way analysis of variance)

Urate transporters regulate intracellular urate accumulation; two of the family members, Glut9 and URAT1, regulate serum urate level [33, 34]. To determine whether this applied to intracellular urate concentration in microglia, we measured Glut9 and URAT1 expression in BV2 cells (Fig. 4a). LPS treatment decreased URAT1 expression, which was reversed by urate pretreatment (Fig. 4b). In addition, while intracellular urate level was increased by 22.7% (from 76.94 ± 6.162 nmol/ml to 94.43 ± 6.660 nmol/ml, *p* = 0.1261), 41.6% (from 76.94 ± 6.162 nmol/ml to 109.0 ± 6.665 nmol/ml, *p* = 0.0242) and 57.2% (from 76.94 ± 6.162 nmol/ml to 121.0 ± 5.911 nmol/ml, *p* = 0.0067), respectively, in a dose-dependent manner by urate plus LPS as compared to treatment with LPS alone, this was abolished by pretreatment with PBN, an inhibitor of both Glut9 and URAT1 (Fig. 4c).

We next investigated whether intracellular accumulation of urate was required for its anti-inflammatory effect in microglia. In BV2 cells, compared to LPS alone, when treated with urate, the release of NO and TNF-α was reduced by 33.5% (*p* < 0.0001) and 31.8% (*p* < 0.0001), respectively, and COX-2 expression was decreased by approximately one fold. Compared to treatment of LPS and urate, with PBN treatment, NO and TNF-α release was reversed by 29.6% (*p* < 0.0001) and 33.8% (*p* < 0.0001), and COX-2 expression was upregulated by 0.6-fold (Fig. 4d–f). Similar results were obtained in primary microglia, with PBN pretreatment reversing the effect of urate on NO and IL-1β release and iNOS expression (Fig. 4g–i). Taken together, these results indicated that the anti-inflammatory effect of urate depended on intracellular accumulation of urate uptaken by urate transporters.

### Urate protected DA neurons from neurotoxicity induced by activated microglia

**Fig. 5:**
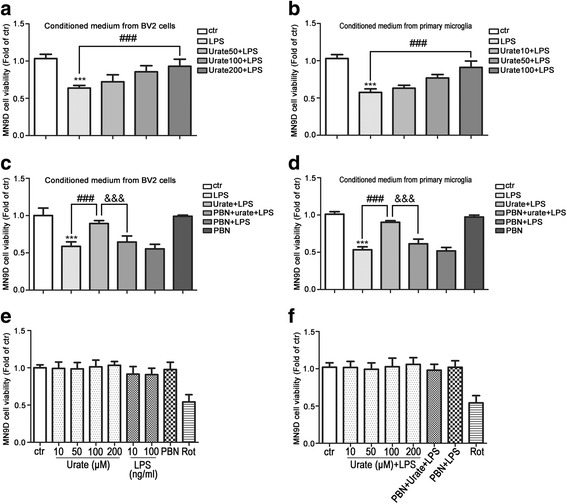
Urate protects DA neurons from neurotoxicity induced by microglia activation. **a**, **b** MN9D cells were incubated for 24 h with conditioned medium from cultures of BV2 cells (**a**, *n* = 4) or rat primary microglia (**b**, *n* = 4) treated with urate plus LPS. Cell viability was measured with the MTS assay. **c**, **d** MN9D cells were incubated for 24 h with conditioned medium derived from cultures of BV2 cells (**c**, *n* = 4) or rat primary microglia (**d**, *n* = 4) treated with LPS, urate+LPS, or PBN+urate+LPS. Cell viability was evaluated with the MTS assay. **e**, **f** MN9D cells were treated with indicated drugs for 24 h, and cell viability was evaluated with the MTS assay (*n* = 3). Rotenone (Rot, 0.5 μM) was used as positive control. Medium from cultures of untreated microglia served as a control (ctr). Data represent the mean ± SD. ^***^p < 0.001 vs. control group; ^###^*p* < 0.001 vs. LPS group; ^&&&^*p* < 0.001 vs. urate+LPS group (one-way analysis of variance)

Microglia activation induces the loss of DA neurons in PD patients. It has also been reported that LPS-induced microglia activation results in damage to DA neurons in vitro [35]. To investigate whether urate protected neurons from the toxic effects of activated microglia, supernatants from cultures of microglia treated with LPS alone or in combination with urate were collected as conditioned medium after measuring the released inflammatory factors (Figs. 1 and 2) and then continued to co-culture with MN9D cells to examine the neuroprotection. Conditioned medium from LPS-treated BV2 cell and primary microglia cultures reduced the viability in MN9D cells, while the conditioned medium from LPS- and urate-treated BV2 cells and primary microglia cultures increased it (Fig. 5a, b). These results suggested that proinflammatory cytokines released into the culture medium by microglia upon LPS stimulation had a negative effect on the viability of MN9D cells, while urate treatment countered these effects.

To confirm the protective effect of urate on neurons, PBN was used to block urate function in microglia and induce proinflammatory cytokine release. Conditioned medium from PBN-pretreated microglia cultures (Fig. 4) reduced the viability in MN9D cells (Fig. 5c, d). However, stimulating MN9D cells under these conditions had almost no effect on cell viability (Fig. 5e, f). Thus, urate protected DA neurons from the toxic effects of activated microglia by inhibiting the release of proinflammatory factors.

### Urate suppressed neuroinflammation induced by activated microglia in an LPS-induced rat PD model

**Fig. 6:**
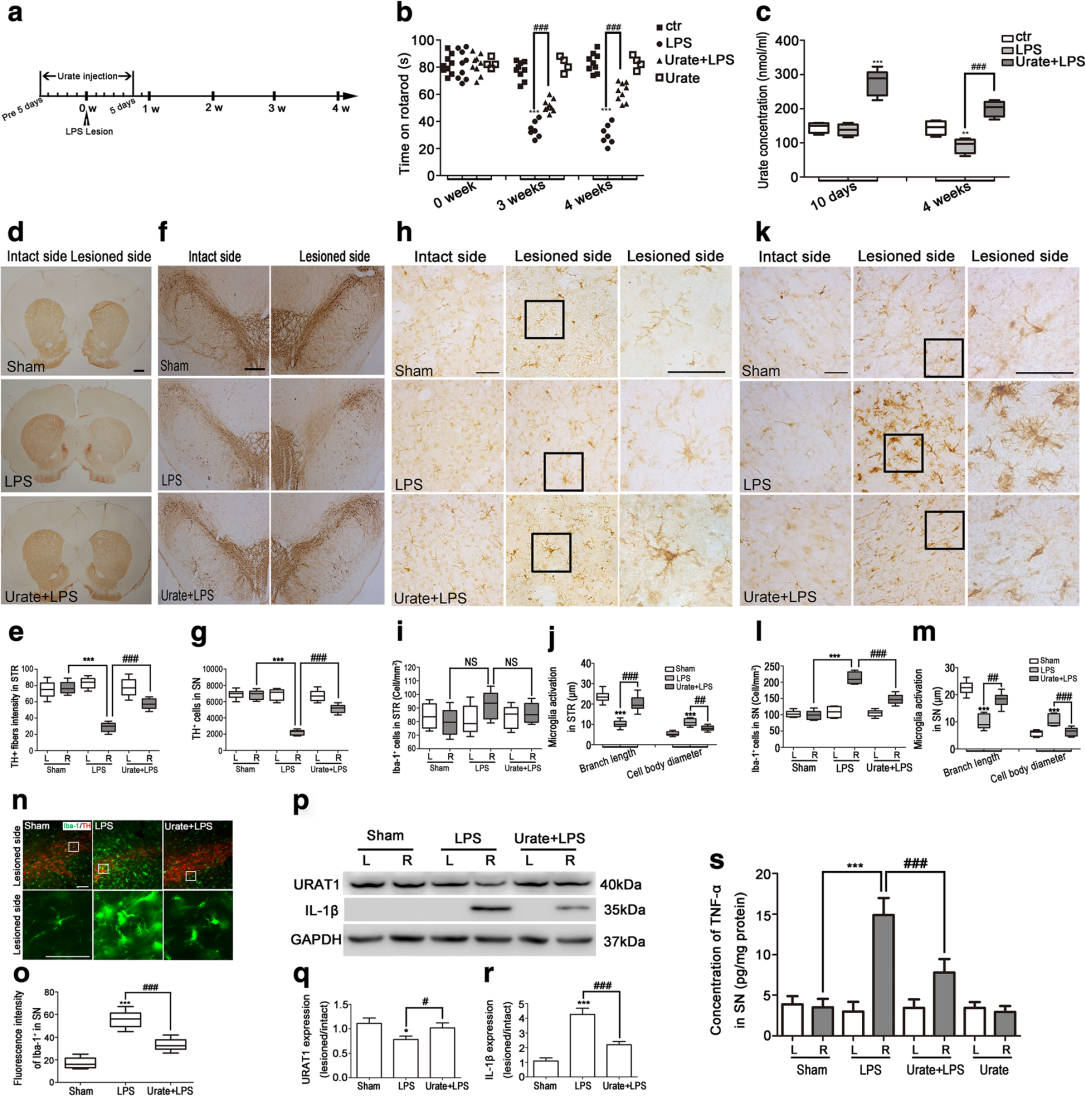
Urate suppresses neuroinflammation induced by activated microglia in a rat model of LPS-induced PD. **a** Schematic illustration of the schedule for urate and LPS administration. **b** Motor coordination was evaluated with the rotarod test at 0, 3, and 4 weeks after LPS injection (*n* = 4–9/group). **c** Plasma concentration of urate was assessed with a fluorometric assay (*n* = 4–9/group). **d**–**g** Representative images of TH immunoreactivity in the STR (**d**, *n* = 4–5/group, scale bar = 1000 μm) and SN (**f**, *n* = 4–5/group, scale bar = 500 μm). Quantitative analysis of TH-positive fibers in STR shown as the intensity of intact side (left, L) and lesioned side (right, R) (**e**). Quantitative analysis of TH-positive cells in SN, shown as the number of TH-positive cells in intact side and lesioned side (**g**). **h**–**m** Representative images of Iba-1 immunoreactivity in the STR (**h**, *n* = 4–5/group, scale bar = 50 μm) and SN (**k**, *n* = 4–5/group, scale bar = 50 μm). Quantitative analysis of Iba-1-positive cells in STR (**i**) and SN (**l**), shown as the number of cells per square millimeter in intact side and lesioned side, respectively. Quantitative analysis of branch length and cell body diameter of Iba-1-positive cells, shown as the absolute of branch length and cell body diameter lesioned side in STR (**j**) and SN (**m**). **n**, **o** Representative images of immunofluorescence analysis of TH-positive (red) and Iba-1-positive (green) cells in the lesioned side of SN (**n**, *n* = 4–5/group, scale bar = 100 μm). Quantitative analysis of fluorescence intensity of Iba-1-positive cells in lesioned side (**o**). Quantification of TH-positive cells in SN was counted by sterology using Stereo Investigator software. TH-positive fibers in STR and Iba-1 positive cells in the STR and SN were performed using Image Pro Plus v5.0 image analysis software. **p**–**r** Protein expression in SN was assessed in animals subjected to the indicated treatments (*n* = 4–5/group). Tissue lysates were analyzed for expression of URAT1 and IL-1β by Western blotting (**p**). The levels were normalized to GAPDH and quantified as the ratio of the lesioned side to the intact side (**q**, **r**). **s** Tissue lysates were prepared and TNF-α concentration of SN was assessed by ELISA (*n* = 4–5/group). Rats injected with vehicle served as the sham. Data represent the mean ± SD. ^*^p < 0.05, ^**^p < 0.01, ^***^p < 0.001 vs. sham group; ^#^*p* < 0.05, ^##^*p* < 0.01, ^###^*p* < 0.001 vs. LPS group (one-way analysis of variance)

To evaluate the effect of urate on microglia activation in vivo, LPS was used to establish a model of neuroinflammation-induced PD. Rats were injected with urate at doses of 200 mg/kg twice daily for five consecutive days before and after LPS injection (Fig. 6a) and subjected to behavioral testing 3 and 4 weeks after LPS injection. LPS-injected rats showed a reduced time on the rotarod, indicating a decline in motor coordination and fatigue resistance. This decrease was abolished by urate administration, and urate alone did not exert any effect (*p* = 0.7579) (Fig. 6b). Plasma urate level was markedly increased after ten consecutive days of urate injection; interestingly, the level of urate was decreased by 35.6% (from 144.1 ± 9.022 nmol/ml to 92.27 ± 10.96 nmol/ml, *p* = 0.0078) in LPS-treated rats compared to the sham group after 4 weeks of LPS injection but was upregulated by approximately 1.2-fold (*p* = 0.0002) with urate administration compared to LPS injection (Fig. 6c). An immunohistochemical analysis revealed that LPS administration reduced the intensity of TH-positive fibers in striatum by 63.6% (*p* < 0.0001) and the number of TH-positive neurons in SN by 67.6% (*p* < 0.0001) on the lesioned side, respectively, which was increased by 96.7% (*p* < 0.0001) and 56.5% (*p* < 0.0001) with urate pretreatment, respectively (Fig. 6d–g).

To evaluate the effect of urate on LPS-induced microglia activation in vivo, we analyzed the expression of the microglia marker, Iba-1, by immunohistochemistry and immunofluorescence analyses. LPS caused changes in the morphology of microglia from a ramified to an amoeboid shape, consistent with their activation. The branches lenght of microglia was reduced approximately from 23.59 ± 0.5994 μm to 11.03 ± 0.4775 μm (*p* < 0.0001), and cell body diameter was increased from 5.11 ± 0.2511 μm to 11.09 ± 0.4491 μm (*p* < 0.0001) in striatum, while urate treatment reversed it from 11.03 ± 0.4775 μm to 20.26 ± 0.8986 μm (*p* < 0.0001) and from 11.09 ± 0.4491 μm to 8.94 ± 0.3362 μm (*p* < 0.0001), respectively. However, there was no obvious microglia number changes in the LPS-lesioned side compared to the intact side (*p* = 0.0523) in STR, which is consistent with previous the report [36] (Fig. 6h–j). Similarly, LPS injection reduced the length of branches of microglia and increased cell body diameter in SN, which was reversed by urate treatment. Meanwhile, the number of Iba-1-positive microglia was observed to decrease by 30.4% (*p* = 0.0003) on the lesioned side of the SN in rats treated with LPS and urate as compared to those treated with LPS only (Fig. 6k–m). Moreover, LPS injection increased the fluorescence intensity of Iba-1 in the SN, which was immunostained with TH. Urate treatment attenuated the effect (Fig. 6n, o). In addition, western blotting analysis showed that LPS decreased URAT1 expression and increased expression of IL-1β in the LPS-injected side of the SN, and these effects were inhibited by urate (Fig. 6p–r). LPS injection induced a 4.3-fold increase of TNF-α by ELISA assay (*p* < 0.0001), while treatment with urate significantly decreased the concentration of TNF-α by 47.7% (*p* < 0.0001, Fig. 6s). The above results indicated that urate suppressed neuroinflammation induced by activated microglia in an LPS-induced rat PD model.

## Discussion

Many risk factors contribute to PD progression, including oxidative stress and neuroinflammation. Epidemiological and clinical studies have reported a correlation between high urate level and a lower risk of developing PD along with a decreased rate of disease progression [12, 37]. H_2_O_2_-induced DA cell death was found to be suppressed by glutathione released by urate-treated astrocytes [22, 38]; moreover, urate can form a complex with iron, which boosts its antioxidant effect [39]. Oxidative stress is closely linked to other components of neurodegenerative process such as nitric oxide stress and inflammation that contribute to neurodegeneration [40]. Reactive oxygen species activate microglia and increase proinflammatory cytokine release; in turn, activated microglia and proinflammatory cytokines perpetuate oxidative stress [40]. The above evidence suggests that urate may exert neuroprotection via inhibiting oxidative stress and also suppressing neuroinflammation in PD. Indeed, we found that urate blocked LPS-induced microglia activation in vitro and in vivo. Intracellular urate accumulation was required for its anti-inflammatory effects and protected DA neurons from neurotoxicity induced by microglia activation. The approach that elevating serum urate level by oral inosine, a urate precursor, was indicated generally safe, tolerable, and effective in early PD, which was proposed to become a potential therapy for PD [41, 42]. Therefore, our findings contribute to the role of urate in anti-inflammation apart from its function on inhibiting oxidative stress [15, 18], which potentially provide a better understanding of controlling these two risk factors for therapeutic treatment in PD.

Microglia secrete proinflammatory cytokines such as NO, TNF-α, and IL-1β upon LPS stimulation [43, 44]. Here, we found that urate inhibited the release of inflammatory factors including NO, TNF-α, and PGE2 while slightly increasing the levels of the anti-inflammatory factors, IL-10 and TGF-β1, in LPS-treated BV2 cells and rat primary microglia. Urate also transformed microglia from an activated to a resting state. These results indicated that urate acted by suppressing the release of proinflammatory factors by activated microglia. Toll-like receptor (TLR) 4 is highly expressed in microglia [45–47], and the activation of TLR4/nuclear factor (NF)-κB signaling can induce microglia activation [48, 49]. Dual-specificity tyrosine phosphorylation-regulated kinase (Dyrk)2 has been shown to increase p65, Akt, and p38 mitogen-activated protein kinase phosphorylation to regulate the release of proinflammatory cytokines in LPS-stimulated BV2 cells [50]. Moreover, the glucagon-like peptide (GLP)-1 receptor/cyclic (c)AMP/protein kinase A/p380205/cAMP response element binding protein signaling pathway was shown to mediate anti-inflammation cytokine production in microglia. Thus, the anti-inflammatory effects of urate may be mediated primarily via TLR4/NF-κB and Dyrk2/AKT signaling and to a lesser degree via GLP-1 receptors.

**Fig. 7:**
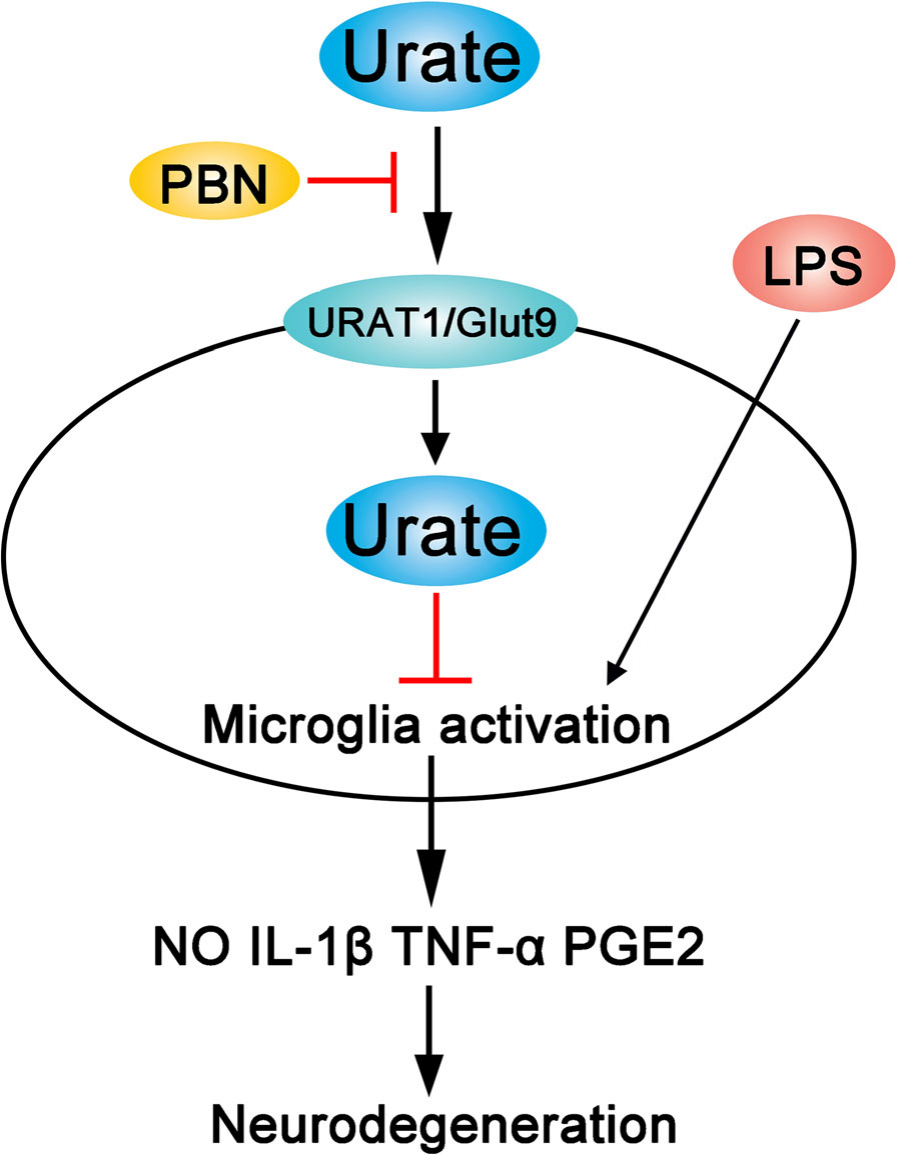
Schematic illustration of the suppression of microglia activation and neuroinflammation by urate in an LPS-induced model of PD. High intracellular levels of urate in microglia protect DA neurons by inhibiting the release of proinflammatory factors

High plasma urate concentration can decrease the risk of PD [51, 52]. Urate level is lower in the SN and cerebrospinal fluid of PD patients as compared to non-PD subjects [1, 53]. Higher serum and cerebrospinal fluid urate concentrations at baseline are associated with slower rates of clinical decline in PD [54]. Thus, urate is considered as a potential diagnostic and prognostic biomarker of PD [12, 13]. Here, we provided the experimental evidence that increased intracellular levels of urate suppress inflammation that can be disturbed by blocker of urate transporters, suggesting that urate elevation may be beneficial for the treatment of neurological disorders including PD. Proinflammatory cytokines released by activated microglia induce neurodegeneration in PD [35]. We found that increased intracellular level of urate in microglia protects DA neurons from microglia activation by reducing the release of proinflammatory factors via urate transporters (Fig. 7). In an LPS-induced rat PD model, urate suppressed microglia activation in the SN and striatum and reversed the decrease in TH-positive neurons. However, treatment of urate and LPS directly affects neurons might not be excluded in vivo. Recent paper has shown that microglia emerge as central players in brain disease [55]. Microglia activation also triggers neurotoxic reactive astrocytes [56] and excitatory neurotransmission mediated by astrocytes [57]. As TLR4 is highly expressed in microglia [46], it is possible that these cells are the major target of the anti-inflammatory effects of urate. Interestingly, a recent study showed that lithium inhibited LPS-induced TLR4 expression and astrocyte activation [58], suggesting that urate may also directly regulate astrocyte activation to mediate neuronal survival. Of note, since chloral hydrate was shown to reduce the inflammation of LPS-induced acute lethal liver injury [59], it might cause less inflammation in LPS-induced PD model for our study, and an alternative method such as pentobarbital [60] might be preferred to be used for anesthesia in the future.

## Conclusion

In summary, we found that urate protected DA neurons from inflammation induced by activated microglia in an LPS-induced PD model, possibly by increasing intracellular levels of urate. As mentioned, in this study, we applied BV2 cells, primary microglia, and the animal model of PD, which are very different from adult microglia in human as their gene expression can diverge significantly [61]. Also, the expression of several inflammatory mediators in BV2 cells may be different from primary microglial cells [62, 63], which might cause an increased inflammation with urate treatment alone in BV2 cells (Fig. 1). Despite these limitations, the study provided evidence for urate inhibiting microglia activation to protect neurons in PD and supported its further clinical development.

## Additional files

Additional file 1: **Figure S1.** BV2 cell viability was detected by MTS assay. BV2 cells were treated with different drugs at various concentrations for 24 h, and cell viability was detected. Untreated cells served as a control over treatments (ctr). NaOH, solvent of urate, and PBN as a blank control. Rotenone (Rot, 0.5 μM) was used as a positive control of cell viability assay. Data represent the mean ± SD (*n* = 3). NS, not significant. ^***^p < 0.001 vs. control group (one-way analysis of variance). (TIF 559 kb)

Additional file 2: **Figure S2.** Primary microglia viability was detected by MTS assay. Primary microglia was treated with different drugs at various concentrations for 24 h, and cell viability was detected. Untreated cells served as a control over treatments (ctr). NaOH, solvent of urate, and PBN as a blank control. Rotenone (Rot, 0.5 μM) was used as a positive control of cell viability assay. Data represent the mean ± SD (n = 3). NS, not significant. ^***^p < 0.001 vs. control group (one-way analysis of variance). (TIF 569 kb)

## Abbreviations

COX: Cyclooxygenase
DA: Dopaminergic
GAPDH: Glyceraldehydes 3-phosphate dehydrogenase
Iba: Ionized calcium-binding adaptor molecule
IL: Interleukin
iNOS: Inducible nitric oxide synthase
LPS: Lipopolysaccharide
NO: Nitric oxide
PBN: Probenecid
PD: Parkinson’s disease
PG: Prostaglandin
SN: Substantia nigra
TH: Tyrosine hydroxylase
TNF: Tumor necrosis factor
