# Supplementary figures and images for: Urate inhibits microglia activation to protect neurons in an LPS-induced model of Parkinson’s disease
Source: J Neuroinflammation. 2018 May 2;15:131. doi: 10.1186/s12974-018-1175-8 (PMC5932803; doi:10.1186/s12974-018-1175-8)

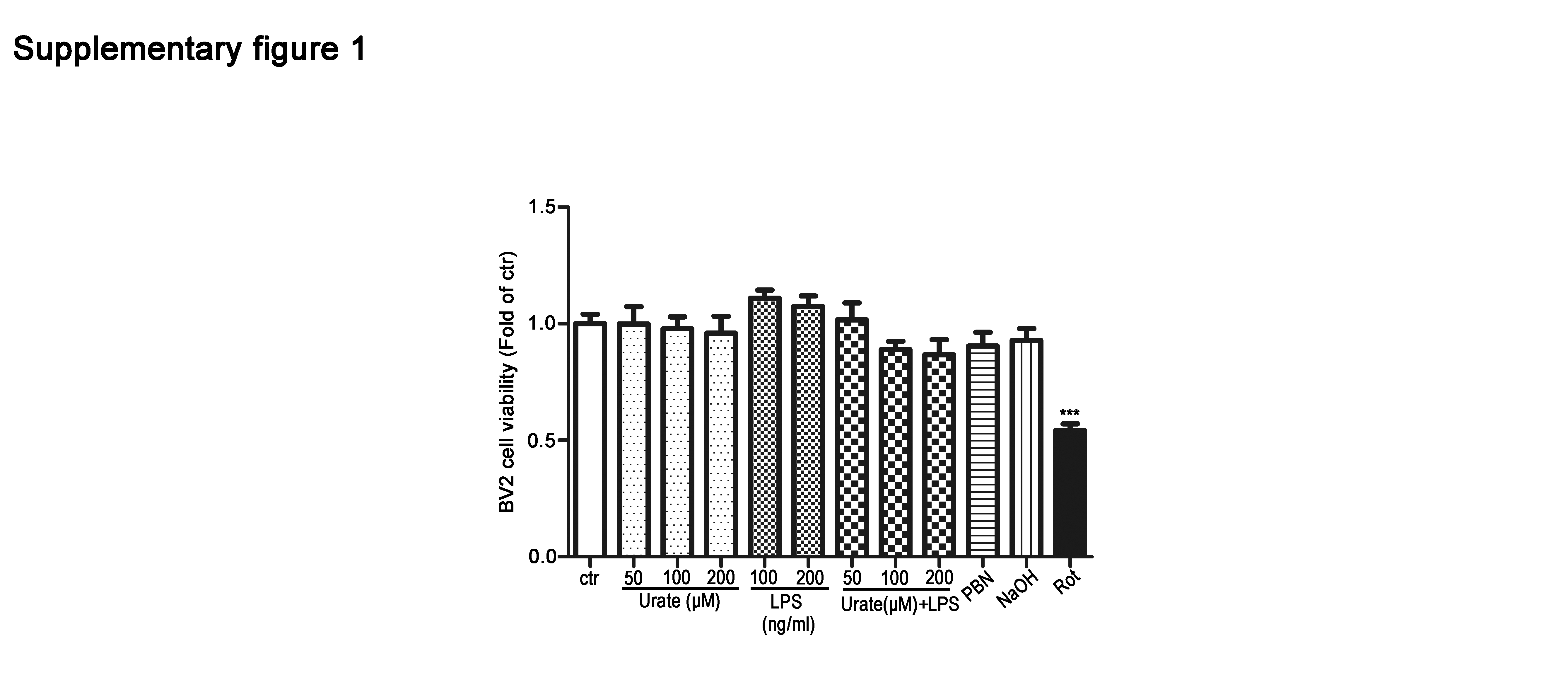

Supplement: Supplementary file 1 — Figure S1. BV2 cell viability was detected by MTS assay. BV2 cells were treated with different drugs at various concentrations for 24 h, and cell viability was detected. Untreated cells served as a control over treatments (ctr). NaOH, solvent of urate, and PBN as a blank control. Rotenone (Rot, 0.5 μM) was used as a positive control of cell viability assay. Data represent the mean ± SD (n = 3). NS, not significant. ***p < 0.001 vs. control group (one-way analysis of variance). (TIF 559 kb) [file 12974_2018_1175_MOESM1_ESM.tif]

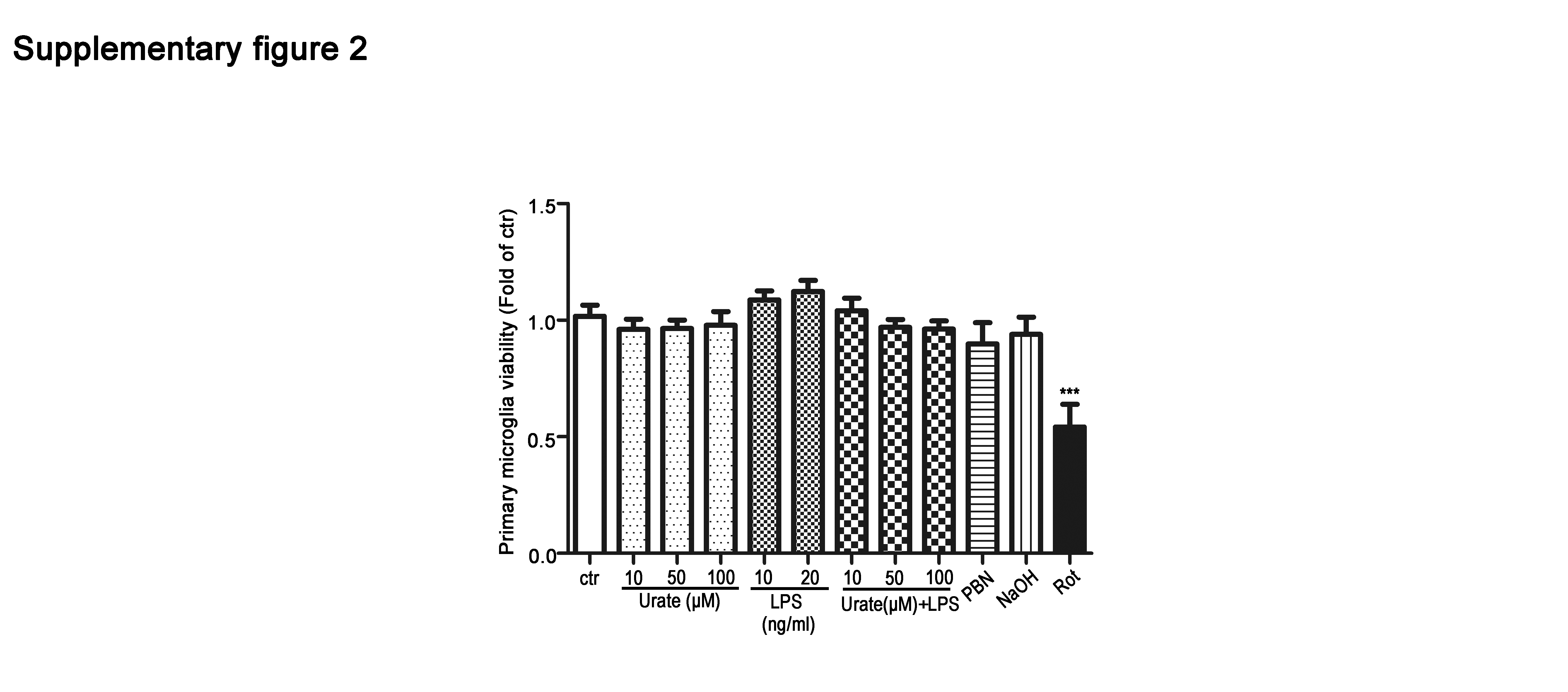

Supplement: Supplementary file 2 — Figure S2. Primary microglia viability was detected by MTS assay. Primary microglia was treated with different drugs at various concentrations for 24 h, and cell viability was detected. Untreated cells served as a control over treatments (ctr). NaOH, solvent of urate, and PBN as a blank control. Rotenone (Rot, 0.5 μM) was used as a positive control of cell viability assay. Data represent the mean ± SD (n = 3). NS, not significant. ***p < 0.001 vs. control group (one-way analysis of variance). (TIF 569 kb) [file 12974_2018_1175_MOESM2_ESM.tif]
